# The Fate of Mitral Valve Regurgitation After TAVI

**DOI:** 10.3390/jcm15218259

**Published:** 2026-10-23

**Authors:** Karin Steiner, Melchior Burri, Nina Feirer, Tobias Rheude, Hendrik Ruge, Markus Krane, Keti Vitanova

**Affiliations:** 1 Department of Cardiovascular Surgery, TUM University Hospital German Heart Center Munich, Technical University Munich, 80636 Munich, Germany; steinerk@dhm.mhn.de (K.S.); feirer@dhm.mhn.de (N.F.); ruge@dhm.mhn.de (H.R.); krane@dhm.mhn.de (M.K.); 2 Department of Cardiovascular Surgery, INSURE (Institute of Translational Cardiac Surgery), TUM School of Medicine and Health, Technical University Munich, 80636 Munich, Germany; 3 Department of Cardiology, TUM University Hospital German Heart Center Munich, Technical University Munich, 80636 Munich, Germany; rheude@dhm.mhn.de; 4 DZHK (German Center for Cardiovascular Research) Partner Site Munich Heart Alliance, 80636 Munich, Germany

**Keywords:** TAVI, aortic stenosis, severe mitral regurgitation, improvement, survival

## Abstract

**Background:** Outcomes of patients undergoing transcatheter aortic valve implantation (TAVI) for severe aortic stenosis with concomitant severe mitral regurgitation (MR) remain incompletely defined. This study aimed to quantify the evolution of high-grade MR after TAVI, compare changes using MR etiology, and assess associations between MR etiology, early improvement, or residual discharge MR and mortality. **Methods:** In a retrospective single-center cohort, 112 TAVI patients with baseline more-than-moderate MR were included (functional *n* = 94; degenerative *n* = 18). MR grade was assessed pre-TAVI, at discharge, and at last available follow-up (serial studies up to ≥5 years), and 5-year all-cause mortality was analyzed. **Results:** At discharge, 34% of patients improved by more than one MR grade, and 26% by one grade. Among 45 patients with available late echocardiographic follow-up, 62% improved by more than one grade, 24% by one grade. Exploratory five-year survival analyses did not demonstrate a significant difference for MR etiology (*p* = 0.23), discharge MR grade (*p* = 0.11), or MR improvement status (*p* = 0.12). **Conclusions:** Among patients surviving to longitudinal echocardiographic follow-up, MR reduction was frequently observed after TAVI. Exploratory analyses did not demonstrate statistically significant differences in 5-year survival according to MR etiology, early MR improvement, or discharge MR severity. However, limited sample size and incomplete follow-up preclude definitive prognostic conclusions. Overall, these findings are compatible with a surveillance-first management, and with individualized consideration of adjunctive mitral therapy for persistent higher-grade symptomatic MR.

## 1. Introduction

Severe aortic stenosis (AS) frequently coexists with mitral regurgitation (MR), creating complex bivalvular interactions that aggravate disease burden, distort hemodynamic assessment, and complicate treatment sequencing [1]. In this setting, altered loading conditions, annular dilatation, and subvalvular remodeling can amplify MR or mask its severity, while MR can reduce forward flow and contribute to low-flow AS physiology [1,2].

In clinical practice, MR is reassessed after transcatheter aortic valve implantation (TAVI), as afterload relief and reverse remodeling may attenuate functional MR and clarify the need for mitral therapy [3,4,5,6]. Concomitant MR often improves after TAVI, but the extent and durability are strongly pathomechanism-dependent. Functional MR, particularly the atrial type, regresses more frequently than degenerative MR, while baseline chamber metrics and mitral apparatus disease are the strongest determinants of improvement [7,8,9,10]. Whether MR improvement translates into better survival is debated: Persistent moderate-to-severe MR is generally associated with higher mortality and heart-failure readmissions, yet causality is uncertain and may vary by etiology and competing risks [10,11,12,13]. Nonetheless, residual MR at follow-up appears to be an important risk marker, and regression after TAVI is linked with improved outcomes [9,10,12,14]. Consequently, contemporary studies advocate individualized, staged strategies with precise MR phenotyping to guide expectations and management after TAVI [9,13,15,16].

Although larger observational studies and meta-analyses have examined MR after TAVI, patients with baseline higher-grade MR remain comparatively under-represented, and long-term serial echocardiographic data in this selected population are limited [17,18]. Additionally, mechanisms are often pooled, and definitions and timing of MR improvement vary, limiting clinical decision-making and leaving the independent relationship between MR regression and survival unclear [19,20]. To address these gaps, we investigated the fate of more-than-moderate MR after TAVI in a single-center cohort, quantified MR evolution through follow-up, and compared trajectories by MR etiology. We further explored associations between MR etiology, early MR improvement, and residual discharge MR with 5-year all-cause mortality alongside key cardiac comorbidities.

## 2. Materials and Methods

### 2.1. Study Design

We conducted a retrospective, single-center cohort study of patients undergoing TAVI for severe AS with concomitant severe MR between 2007 and 2023. Clinical data, procedural data, and longitudinal follow-up, including serial echocardiography for up to ten years, were obtained from the institutional TAVI database. Eligibility for the study was determined from baseline characteristics and was independent of subsequent echocardiographic follow-up availability. Vital-status follow-up was complete throughout the observational period. All included patients had more-than-moderate MR or severe MR at baseline. All patients underwent interdisciplinary Heart Team evaluation before TAVI. Treatment selection was based on contemporaneous guideline recommendations and incorporated calculated surgical risk, age, comorbidity burden, overall clinical condition, anatomical and procedural characteristics, concomitant valvular disease, and the anticipated risks and benefits of surgical versus transcatheter treatment.

TAVI was not performed as part of a prospectively predefined staged aortic–mitral intervention strategy. Patients with persistent clinically relevant MR underwent repeated clinical and echocardiographic reassessment, and subsequent mitral intervention was considered individually. Patients undergoing mitral valve intervention after TAVI contributed echocardiographic data to the analysis only until the date of the index mitral intervention. Subsequent mitral intervention did not result in censoring from the all-cause survival analysis. Estimated glomerular filtration rate was not reported because the laboratory methodology used for its calculation changed during the extended study period.

Primary echocardiographic outcomes were change in MR grade and MR improvement category at discharge and follow-up. A reduction of exactly one MR category was defined as improvement by one grade, whereas a reduction of two or more categories was defined as improvement by more than one grade. Stable MR category or worsening was defined as no improvement. Key secondary analyses compared these outcomes using MR etiology. Key clinical analyses evaluated the association of five-year survival with MR etiology, discharge MR grade, MR improvement status, NYHA class, baseline left ventricular ejection fraction (LVEF), and tricuspid valve regurgitation (TR). Survival time was calculated from the date of the index TAVI procedure to death or censoring.

### 2.2. Echocardiography Data

Transthoracic echocardiography (TTE) was evaluated at three prespecified time points: baseline (pre-TAVI), discharge (post-procedural), and follow-up (last available at least one year after TAVI). Follow-up examinations were performed according to clinical routine, and not at a uniform prespecified time point. Accordingly, long-term findings represent the last available echocardiographic assessment. MR and TR severity were adjudicated by a comprehensive multiparametric approach (qualitative, semi-quantitative, and quantitative) consistent with contemporary institutional routine, classified with a six-level scale as none, mild, mild-moderate, moderate, moderate–severe, and severe [21].

MR severity was classified on a six-level ordinal scale as none, mild, mild-to-moderate, moderate, moderate-to-severe, and severe.

MR grading was adjusted for survival analysis and categorized as follows: mild–moderate as moderate, moderate–severe as severe. MR etiology was classified as degenerative or functional, using an integrated assessment of valve morphology and the mechanism of regurgitation. Degenerative MR was characterized by intrinsic structural leaflet or chordal abnormalities, whereas functional MR was defined as MR attributable predominantly to ventricular or atrial/annular remodeling. Carpentier leaflet-motion classification was recorded as type I (normal leaflet motion), type II (excessive leaflet motion), type IIIa (restricted motion during systole and diastole), or type IIIb (restricted motion during systole) [22]. Patients were stratified into subgroups based on NYHA class or LVEF (more than 50%, 35–50%, and below 35%) [23,24].

### 2.3. Statistical Analysis

Continuous variables are reported as mean ± standard deviation, and categorical variables are presented as counts and percentages. Within-patient changes for ordinal data between prespecified time points were tested using the Wilcoxon signed-rank test, while continuous data were analyzed using paired *t*-Test. For binary data, McNemar’s test was applied. Time-to-event outcomes were estimated using Kaplan–Meier methods, and between-group differences were assessed by log-rank testing. The significance level was set at *p* < 0.05. Analyses were conducted in Python (3.12; Python Software Foundation, Beaverton, OR 97008, USA).

## 3. Results

### 3.1. Study Cohort

**Figure 1:**
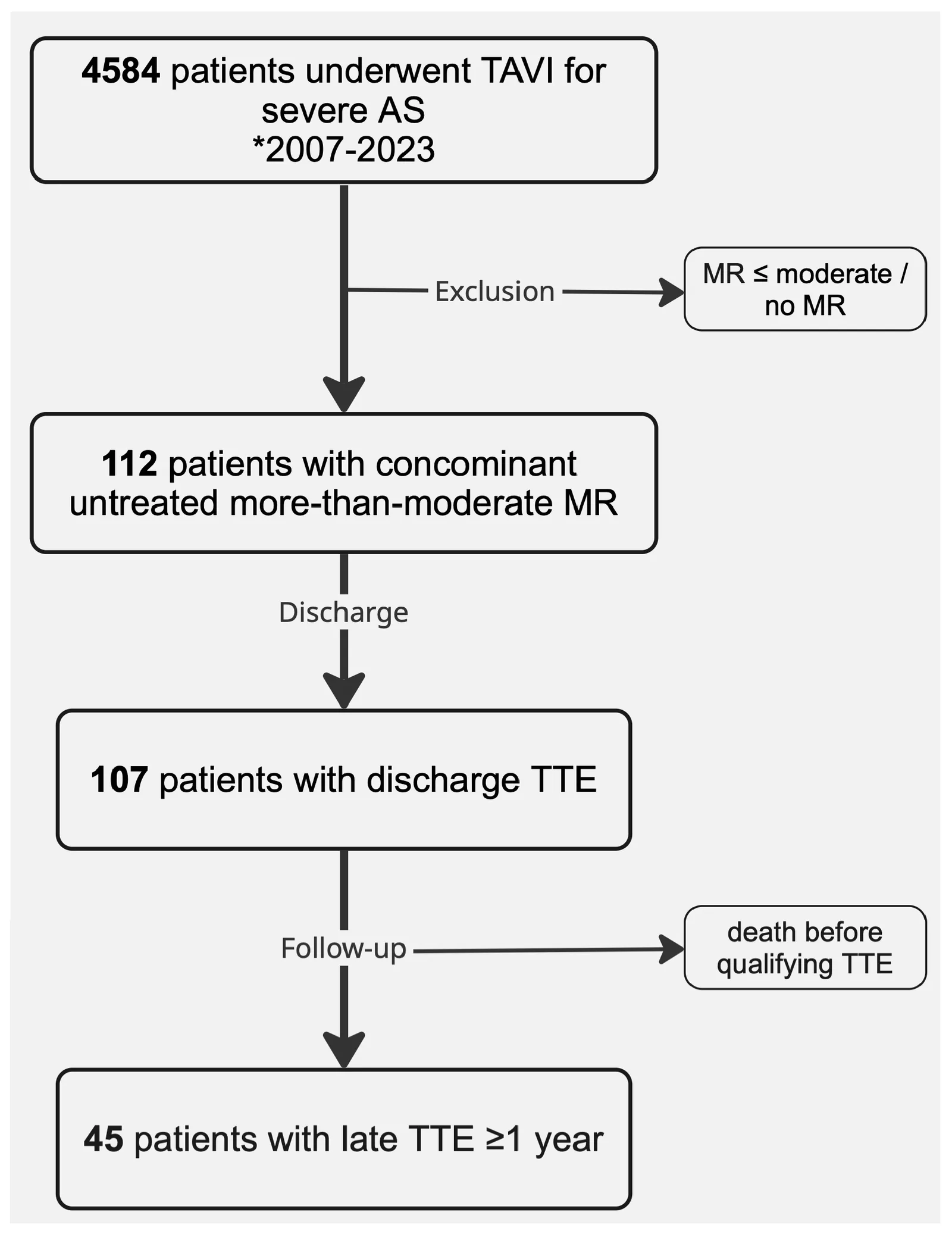
Participant flow chart. Of 4584 patients undergoing TAVI for severe aortic stenosis (AS) between 2007 and 2023, 112 with concomitant mitral regurgitation (MR) were included. Post-procedural transthoracic echocardiogram (TTE) was available in 107 patients and late TTE at ≥1 year in 45, with attrition primarily due to death before qualifying follow-up imaging. * means time sign.

Of the 4584 TAVI patients screened, 112 patients with severe aortic stenosis met the inclusion criteria of more-than-moderate baseline MR (Figure 1).

The study population therefore represented a selected subgroup. Inclusion was independent of concomitant aortic regurgitation, and baseline MR severity was distributed as follows: severe *n* = 50 and moderate–severe *n* = 62 (Table 1). MR mechanism was further characterized according to Carpentier leaflet motion as type IIIa (6 patients), IIIb (83 patients), II (18 patients), and I (5 patients).

### 3.2. Baseline Characteristics

The mean age in this cohort was 82 years, and 56.3% were female (Table 1). Most patients were in NYHA class III (77.7%), with a mean EuroSCORE II of 6.8 and a mean STS of 6.4. Concomitant coronary artery disease (CAD) was present in 50.9%, atrial fibrillation in 49.1%, prior stroke in 14.3%, and prior percutaneous coronary intervention (PCI) in 24.1%. Baseline echocardiographic characteristics showed preserved LVEF > 50% in 50.9%, moderately reduced LVEF 35–50% in 36.6%, and severely reduced LVEF < 35% in 12.5% of patients. Left ventricular end-diastolic diameter (LVEDd) was 50 mm on average. Concomitant tricuspid regurgitation (TR) more than mild was present in 58% of patients.

### 3.3. Post-Procedural Data

Post-procedure echocardiographic assessment was available in 107 patients. There was no significant change in discharge LVEF (*p* = 0.612; Table 1 and Table 2), LVEDd (*p* = 0.521), RV size (*p* = 0.613), AR (*p* = 0.083), or MS (*p* = 0.939) compared to baseline (Table 1 and Table 2). Residual MR was mild in 14%, mild–moderate in 14%, moderate in 22.4%, moderate–severe in 27.1%, and severe in 22.4% of patients, with significant change (*p* < 0.001) compared to baseline (Table 1 and Table 2). Concomitant TR more than mild was present in 38.4%, with a significant change in distribution versus baseline (*p* = 0.020; Table 1 and Table 2).

### 3.4. Follow-Up Data

Late clinical and echocardiographic follow-up was available in 45 patients, with a mean follow-up duration of 4.18 years (SD ± 2.06 years). Vital status was available for all patients throughout the complete observational period from 2007 to 2024. Ten patients required subsequent mitral valve intervention: Mitral Transcatheter Edge-to-Edge Repair (mTEER) was performed in six patients, Transcatheter Mitral Valve Implantation (TMVI) was performed in two patients, and Surgical Mitral Valve Replacement (MVR) was performed in two patients (Table 3). All 112 patients had died by the end of the observational period. In-hospital mortality during the initial hospitalization occurred in 13 patients (11.6%), of whom two patients died on the day of the TAVI procedure. During the total observational period, cardiovascular death was recorded in 18.8% (*n* = 21) and non-cardiovascular death in 43.8% (*n* = 49). Mean time to death was 2.6 years (SD ± 2.7 years).

Mid-term echocardiographic assessment was available in 45 of 112 patients (40.2%), with a mean of 4.18 years. Among these patients, there was a significant change in functional status (*p* = 0.002) compared to baseline, with 31.1% remaining in NYHA class III, and 15.6% in NYHA class IV (Table 1 and Table 4). There was no difference in atrial fibrillation (*p* = 0.228), but a significantly higher number of patients (*p* = 0.005) had a permanent pacemaker (Table 1 and Table 4). LVEF (*p* = 0.782), LVEDd (*p* = 0.673), distribution of MS (*p* = 0.464), AR (*p* = 0.854), or TR (*p* = 0.236) were not significantly different from baseline (Table 1 and Table 4). There was a significant change in RV size (*p* = 0.007) and MR distribution (*p* < 0.001; Table 1 and Table 4).

### 3.5. Evolution of Mitral Valve Regurgitation After TAVI

#### 3.5.1. Distribution of Mitral Valve Regurgitation Severity

**Figure 2:**
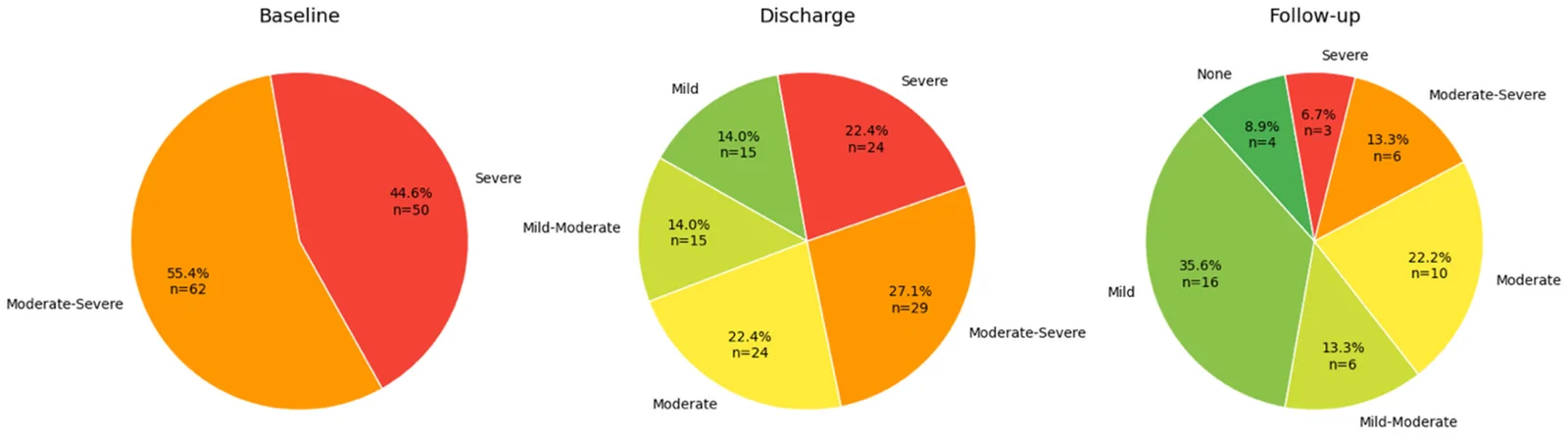
Distribution of mitral regurgitation severity, from baseline preprocedural to hospital discharge and to follow-up. Follow-up is defined as the last echocardiography performed after a period of one year. Annotations with percentages and counts based on available data.

At discharge, MR severity shifted towards mild MR in 14.0% (*n* = 15), mild–moderate MR in 14.0% (*n* = 15), and moderate MR in 22.4% (*n* = 24), while moderate–severe MR (27.1%, *n* = 29) and severe (22.4%, *n* = 24) persisted in a subset of patients (Figure 2). At follow-up, 8.9% (*n* = 4) of patients had no MR, 35.6% (*n* = 16) had mild, 13.3% (*n* = 6) mild–moderate, and 22.2% (*n* = 10) moderate MR. Residual higher-grade MR was present, and was moderate–severe in 13.3% (*n* = 6) and severe in 6.7% (*n* = 3) of patients. A total of 107 patients had evaluable discharge MR, while echocardiographic late follow-up was available in 45 patients.

#### 3.5.2. Distribution of Mitral Valve Regurgitation Severity Based on Etiology

**Figure 3:**
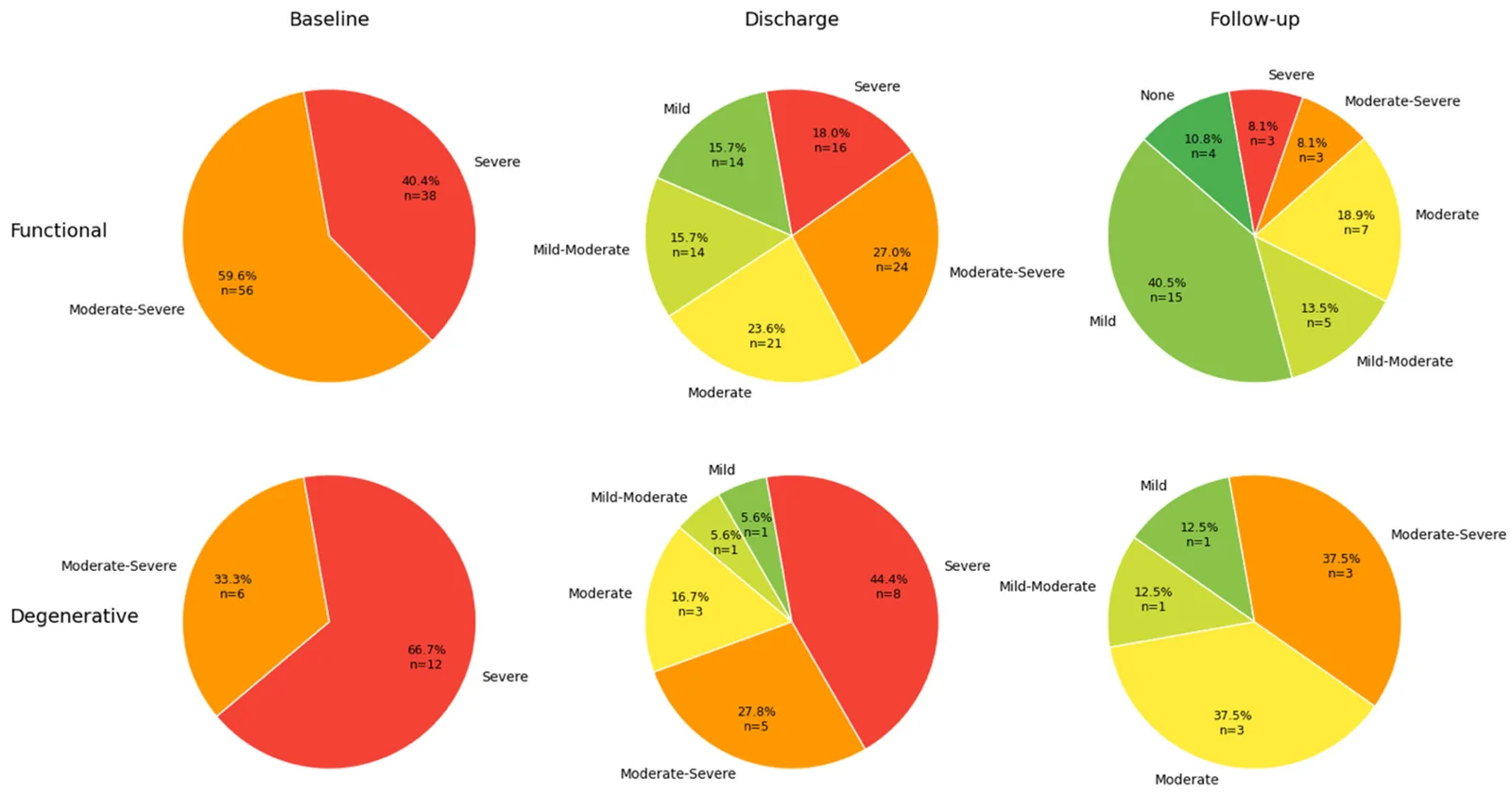
Distribution of mitral regurgitation severity based on etiology, from baseline preprocedural to hospital discharge and to follow-up. Etiology is defined as functional or degenerative. Follow-up is defined as the last echocardiography performed after a period of one year. Annotations with percentages and counts, based on available data.

At discharge, functional MR shifted toward mild MR in 15.7% (*n* = 14), mild–moderate in 15.7% (*n* = 14), and to moderate MR in 23.6% (*n* = 21; Figure 3). In the degenerative MR cohort, one patient (5.6%) had mild MR, and one patient (5.6%) had mild–moderate MR, while three patients (16.7%) had moderate MR at discharge. At follow-up, the functional group showed no MR in 10.8% (*n* = 4), mild in 40.5% (*n* = 15), mild–moderate in 13.5% (*n* = 5), and moderate MR in 18.9% (*n* = 7). In the degenerative MR cohort, mild MR was present in 12.5% (*n* = 1), mild–moderate in 12.5% (*n* = 1), and moderate MR in 37.5% (*n* = 3) at follow-up. Late echocardiographic assessment was available in 45 of 112 patients (40.2%).

#### 3.5.3. Improvement in Mitral Valve Regurgitation

**Figure 4:**
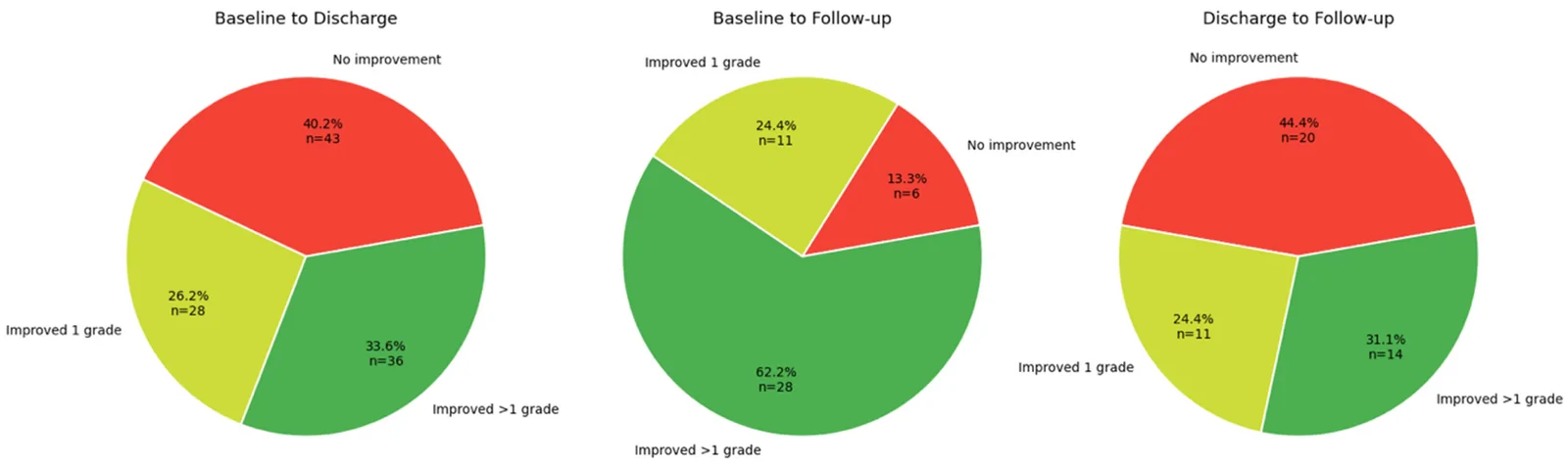
Mitral regurgitation grade improvement, from baseline preprocedural to hospital discharge and to follow-up. Follow-up is defined as the last echocardiography performed after a period of one year. Annotations with percentages and counts, based on available data.

From baseline (pre-procedure) to discharge, MR improved by one grade in 26.2% (*n* = 28) and by more than one grade in 33.6% (*n* = 36), whereas 40.2% (*n* = 43) showed no improvement (Figure 4). Considering the overall change from baseline to follow-up, MR improved in 86.6% of patients, including 24.4% (*n* = 11) with one grade and 62.2% (*n* = 28) with more than one grade reduction of MR. A total of 13.3% (*n* = 6) of patients showed no improvement in MR at follow-up. Mid-term echocardiographic assessment was available in 45 of 112 patients (40.2%).

#### 3.5.4. Improvement in Mitral Valve Regurgitation Based on Etiology

**Figure 5:**
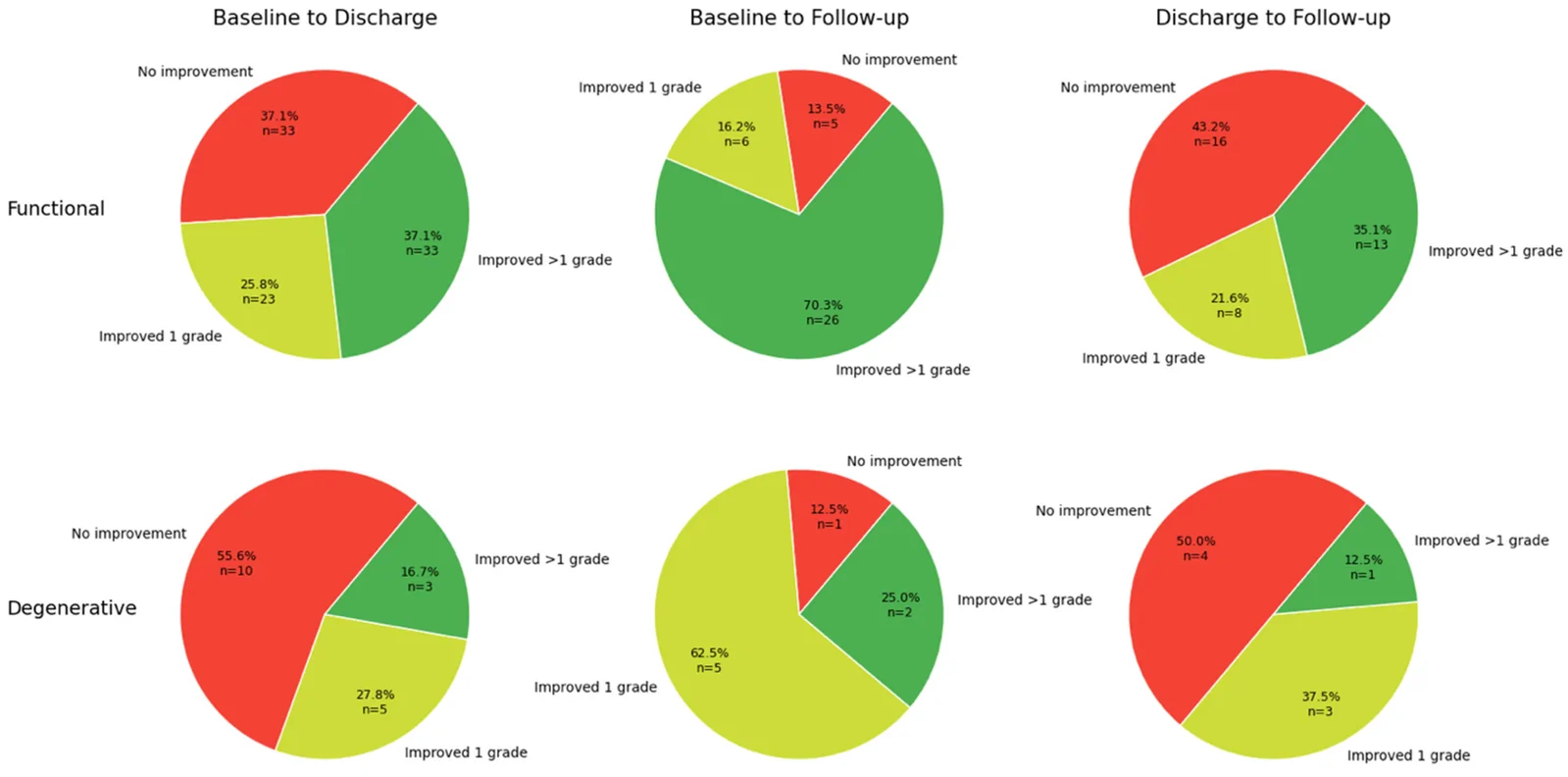
Mitral regurgitation grade improvement stratified by etiology, from baseline preprocedural to hospital discharge and to follow-up. Etiology is defined as functional or degenerative. Follow-up is defined as the last echocardiography performed after a period of one year. Annotations with percentages and counts, based on available data.

From baseline to discharge, functional MR improved by one or more than one grade in 62.9% (one grade 25.8% [*n* = 23], more than one grade 37.1% [*n* = 33]) (Figure 5). Degenerative MR improved by one or more than one grade in 44.5% (one grade 27.8% [*n* = 5], more than one grade 16.7% [*n* = 3]). Considering the net change from baseline to follow-up, functional MR improved in 86.5% (one grade 16.2% [*n* = 6], above one grade 70.3% [*n* = 26]), whereas degenerative MR improved in 87.5% (one grade 62.5% [*n* = 5], above one grade 25.0% [*n* = 2]).

#### 3.5.5. The Fate of Mitral Valve Regurgitation over Time

**Figure 6:**
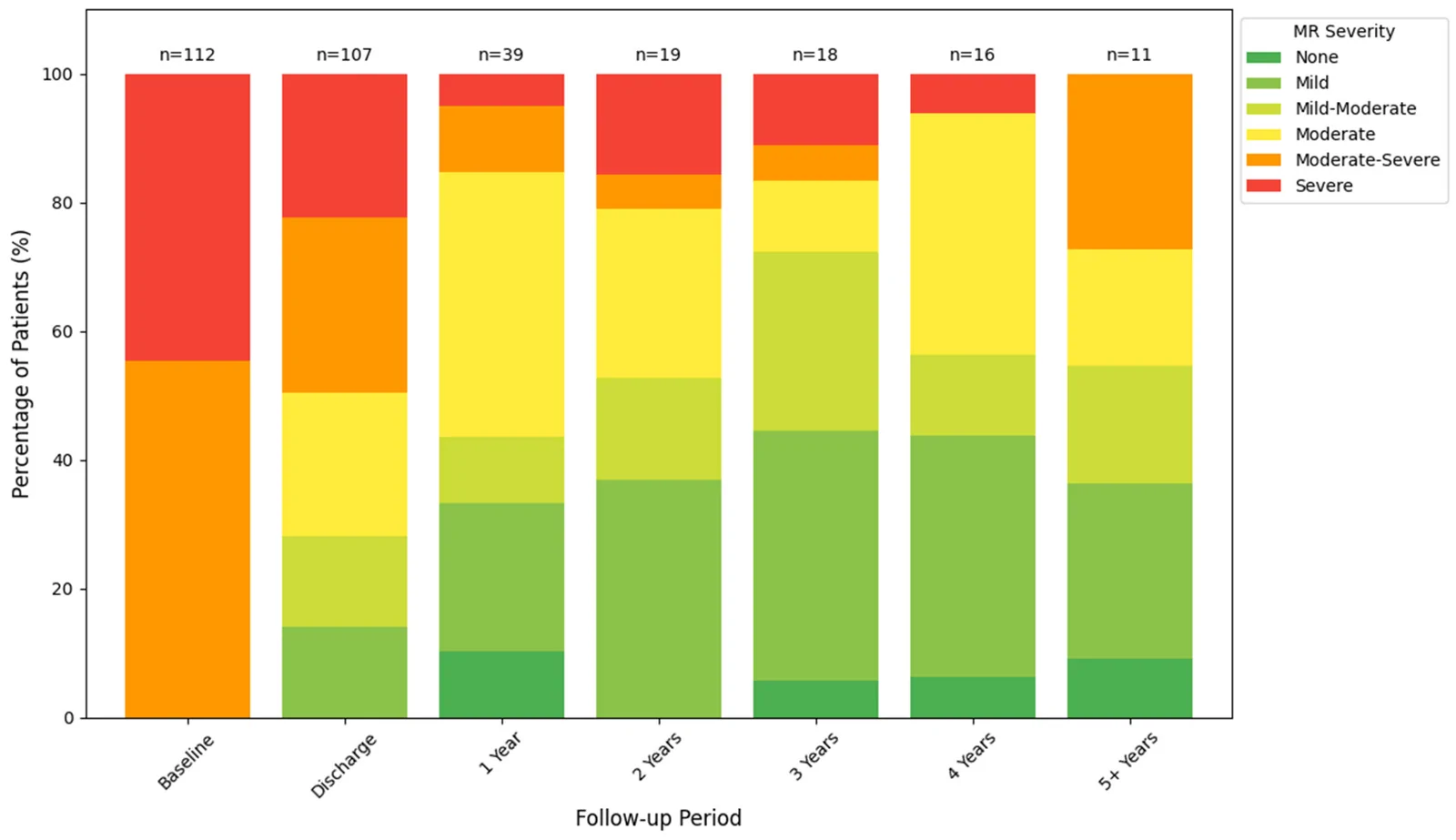
Evolution of mitral regurgitation severity after TAVI. From baseline preprocedural up to five years. Patient numbers represent individuals with available echocardiographic assessment at each time point. Examinations beyond 5 years were combined into 5+ year category.

Across serial echocardiographic assessments over five years after the index procedure, severity shifted from higher-grade MR at baseline toward lower grades after TAVI (Figure 6). Relative to the pre-procedural assessment, MR severity has significantly improved at one year (Wilcoxon *p* < 0.001), two years (*p* < 0.001), three years (*p* = 0.013), and four years (*p* = 0.002), and at TTE performed after five years (*p* = 0.004).

### 3.6. Survival of Patients with Mitral Regurgitation After TAVI

#### 3.6.1. Overall Survival

**Figure 7:**
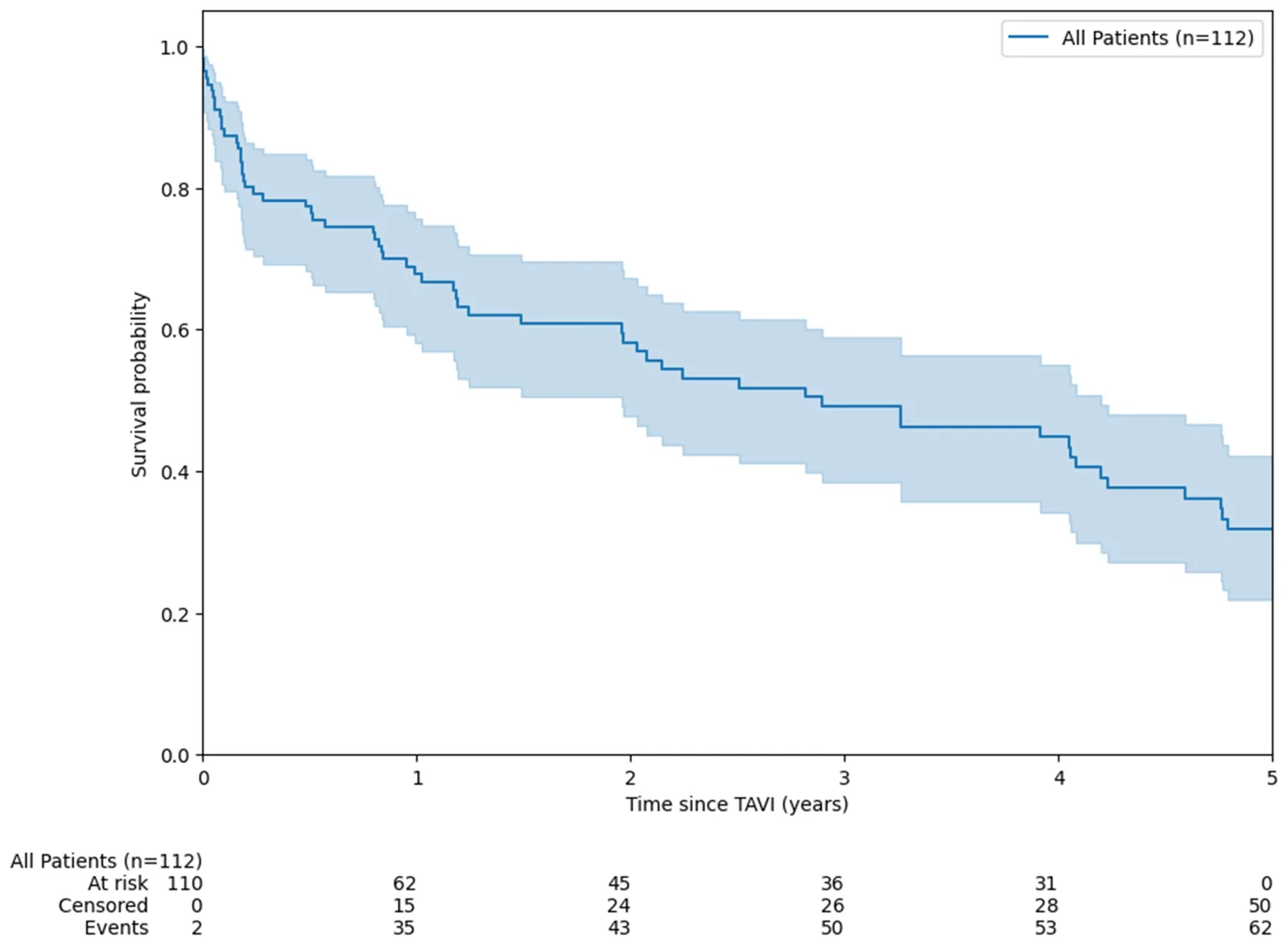
Kaplan–Meier curve of 5-year survival of patients post-TAVI; 95% confidence interval shown as a shaded area, *x*-axis shows years since procedure, *y*-axis shows survival probability.

In the overall cohort, the Kaplan–Meier 5-year estimate demonstrated a continuous, stepwise decline in survival following TAVI (Figure 7). The curve showed a comparatively steeper decrease within the first year, followed by ongoing attrition without evidence of a late-survival plateau.

#### 3.6.2. Survival Estimation Based on Mitral Valve Regurgitation Etiology

**Figure 8:**
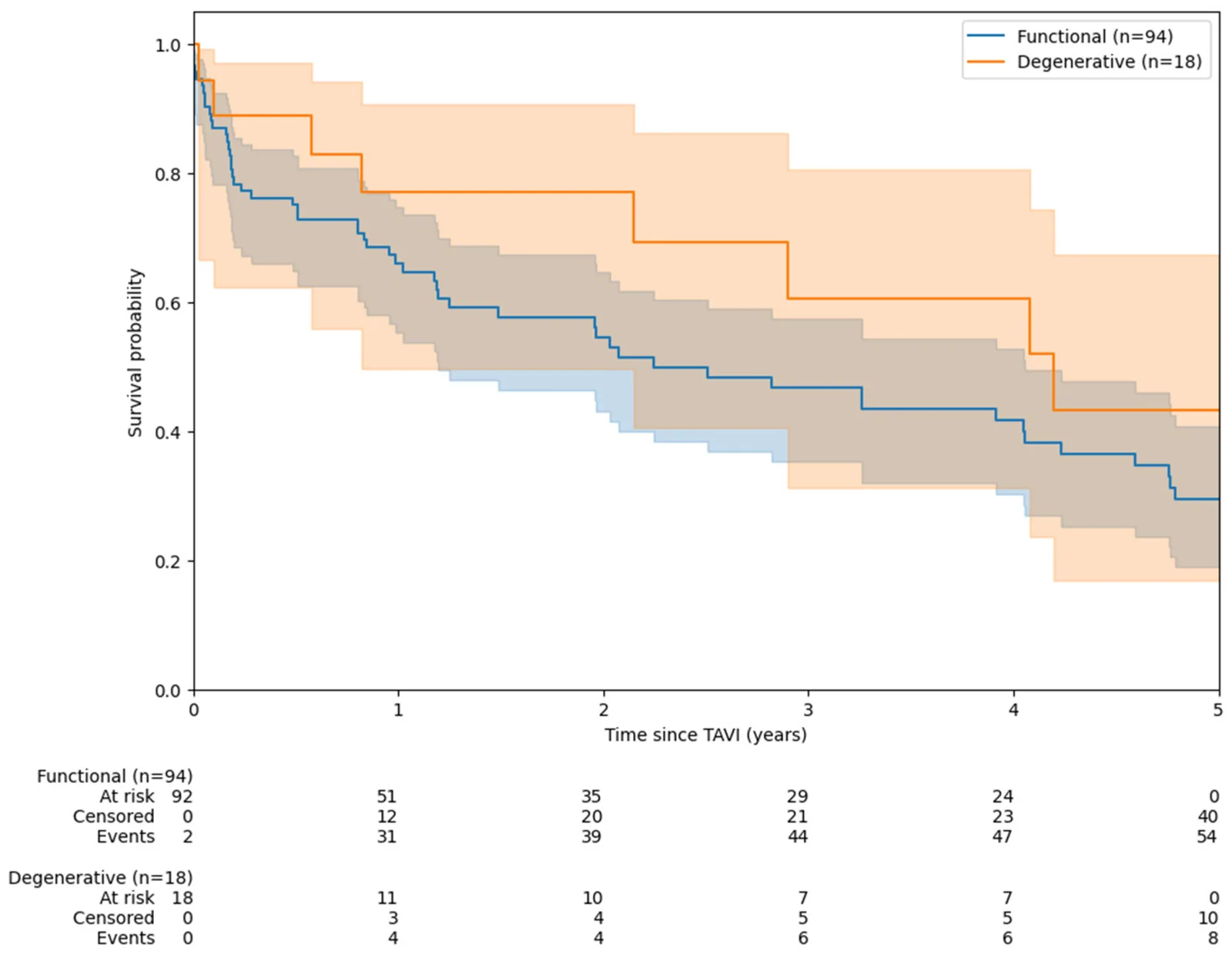
Kaplan–Meier curves comparing 5-year survival based on mitral regurgitation etiology (functional or degenerative); 95% confidence interval shown as a shaded area, *x*-axis shows years since procedure, *y*-axis shows survival probability. Log-rank χ^2^ = 1.43; *p* = 0.23.

Kaplan–Meier five-year survival estimates stratified by MR etiology showed numerically higher survival in the smaller degenerative cohort (*n* = 18) compared with the functional cohort (*n* = 94) (Figure 8). However, exploratory Kaplan–Meier analysis did not demonstrate a statistically significant difference in five-year survival according to MR etiology in log-rank testing (χ^2^ = 1.43; *p* = 0.232).

#### 3.6.3. Survival Estimation Based on Post-Procedure Mitral Valve Regurgitation Severity

**Figure 9:**
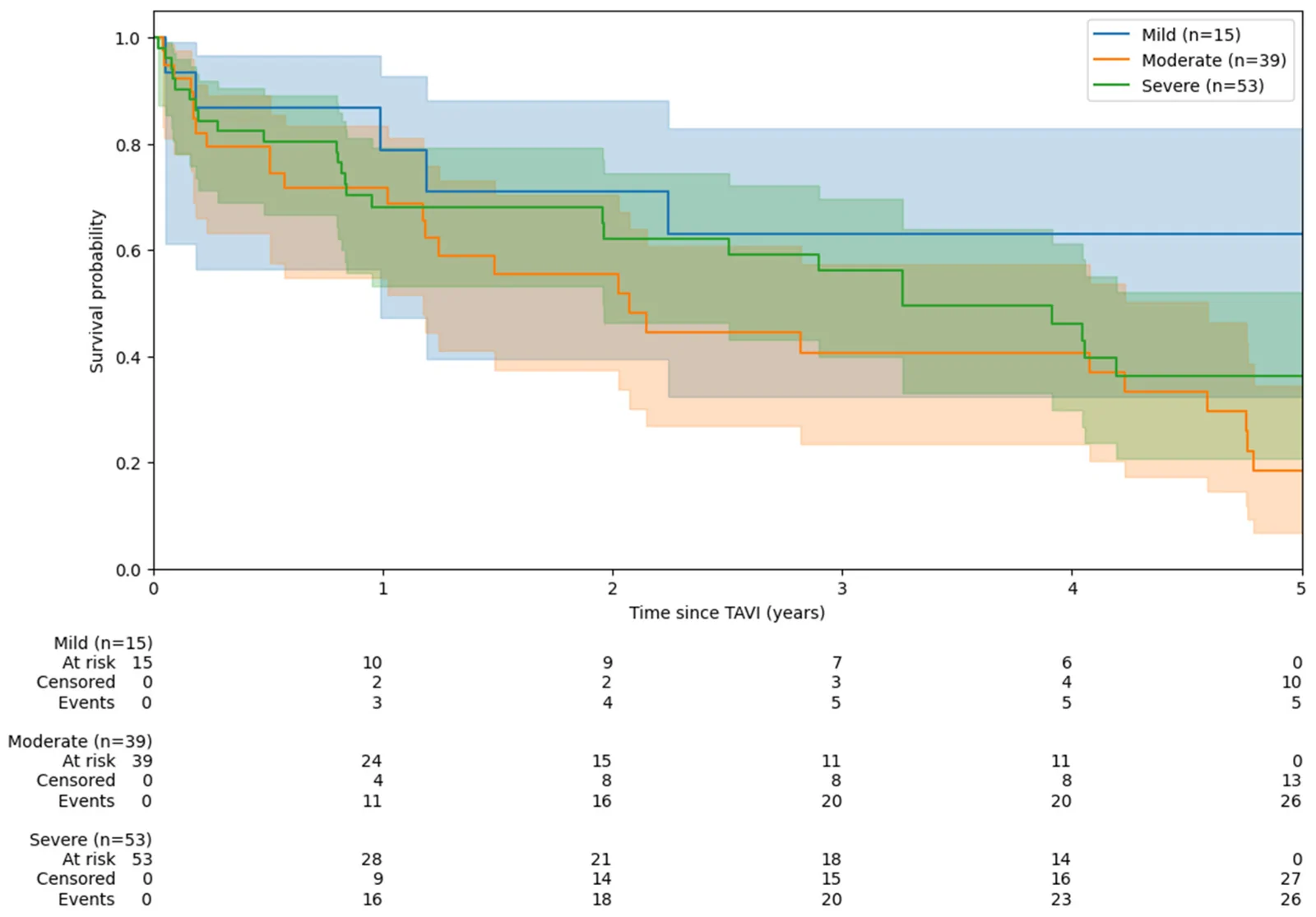
Kaplan–Meier curves comparing 5-year survival based on discharge mitral regurgitation. MR grade at discharge after TAVI was categorized as follows: Mild–Moderate as Moderate, Moderate–Severe as Severe. Shaded areas indicate 95% confidence interval, based on available data. *x*-axis shows years since the procedure, *y*-axis shows survival probability. Log-rank χ^2^ = 4.48, *p* = 0.11.

Kaplan–Meier five-year survival stratified by discharge MR grade (mild, *n* = 15; moderate, *n* = 39; severe, *n* = 53) showed numerically higher survival in patients with mild residual MR (Figure 9). However, the overall comparison across all three severities was not statistically significant (log-rank χ^2^ = 4.48, *p* = 0.11).

#### 3.6.4. Survival Estimation Based on Mitral Valve Regurgitation Improvement

**Figure 10:**
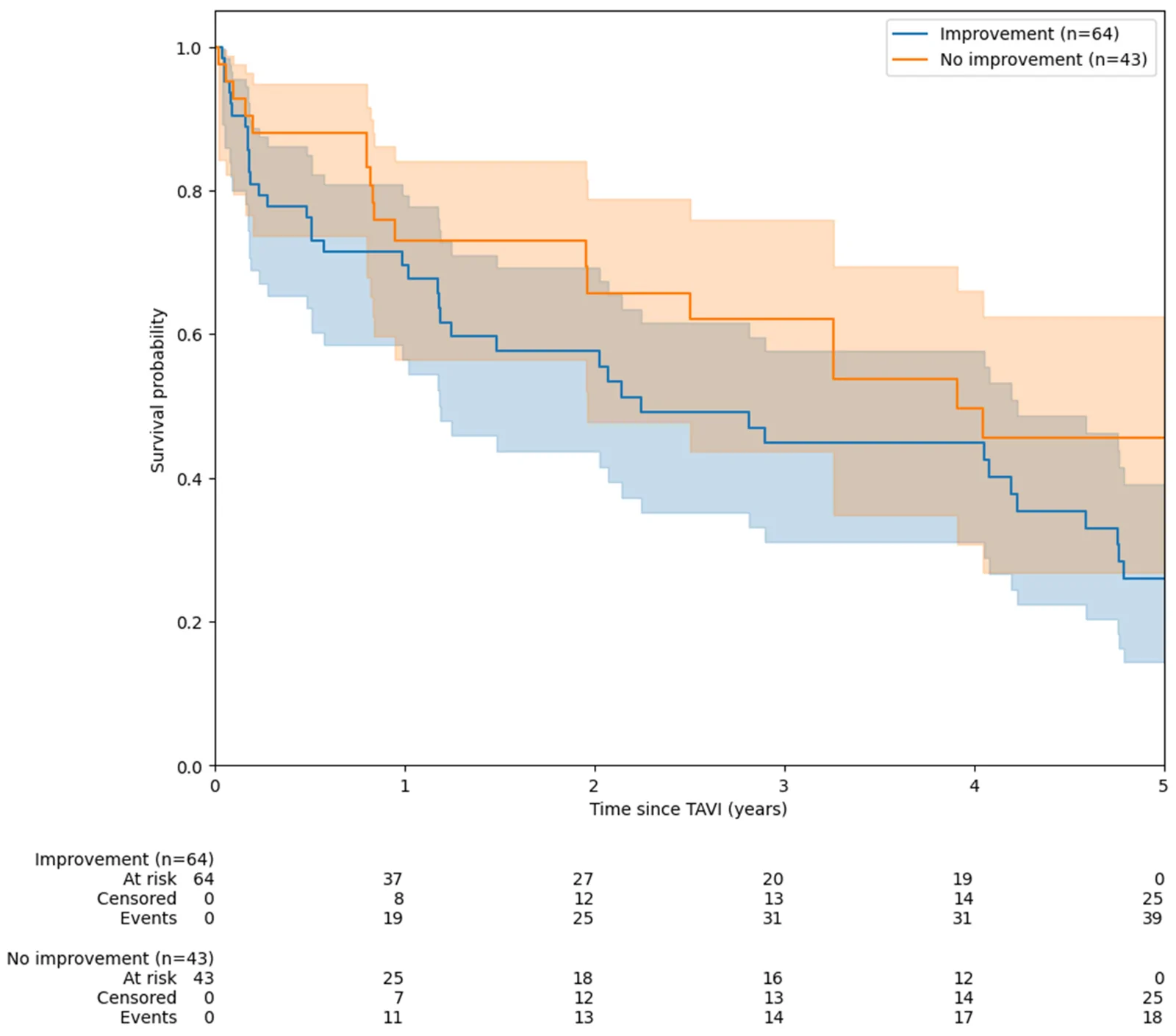
Kaplan–Meier curves comparing 5-year survival based on mitral regurgitation improvement. Improvement status based on discharge MR grade after TAVI. Shaded areas indicate 95% confidence interval, the *x*-axis shows years since the procedure, and the *y*-axis shows survival probability. Log-rank χ^2^ = 2.37, *p* = 0.12.

In Kaplan–Meier survival estimates stratified by MR improvement at discharge after TAVI, the between-group difference was not statistically significant in this exploratory unadjusted analysis (log-rank χ^2^ = 2.37, *p* = 0.123; Figure 10). Thus, this analysis did not demonstrate an association between early MR improvement and 5-year survival. Survival analysis stratified by MR improvement in the functional cohort also showed no statistically significant difference (χ^2^ = 1.37, *p* = 0.243; Supplementary Figure S1). Further Kaplan–Meier analyses showed no significant association of 5-year survival with baseline LVEF impairment (global log-rank χ^2^ = 1.27, *p* = 0.531, Supplementary Figure S2), baseline TR severity (χ^2^ = 0.40, *p* = 0.824; Supplementary Figure S3), or overall comparison of NYHA class (χ^2^ = 5.38, *p* = 0.146), while NYHA IV was associated with worse survival than NYHA III on pairwise comparison (χ^2^ = 4.20, *p* = 0.040; Supplementary Figure S4).

## 4. Discussion

### 4.1. Rationale

Severe AS frequently coexists with MR, complicating grading and treatment sequencing. Although MR frequently improves after TAVI, evidence is heterogeneous and limited by pooled mechanisms, variable definitions, and under-representation of more-than-moderate MR cohorts, limiting clinical decision-making in high-risk subgroups [4,7,17,18]. Consequently, the independent prognostic impact of MR regression remains unclear in the setting of substantial competing risk [7,10,12]. While several larger studies have already evaluated MR evolution and prognosis after TAVI, the essence of the present study is its focus on a selected cohort with baseline higher-grade MR and serial assessment beyond one year. We therefore analyzed a single-center cohort to characterize MR evolution and assess whether MR etiology, early improvement, or residual discharge MR is associated with 5-year mortality.

### 4.2. Main Findings

We observed consistent MR improvement from baseline to discharge and further, to mid-term follow-up, indicating both early hemodynamic effects and continued post-procedural evolution after TAVI. At discharge, MR improved in 59.8% of patients, while MR improved in 86.6% of patients with available follow-up. At late follow-up, any MR improvement was observed with similar frequency in functional and degenerative MR (86.5% vs. 87.5%). However, the magnitude of improvement differed descriptively, with a reduction of more than one grade observed in 70.3% of functional versus 25.0% of degenerative MR cases, consistent with prior evidence [7,8]. Despite improvement, the cohort exhibited substantial early and ongoing mortality over five years. Early MR improvement (*p* = 0.12), MR etiology (*p* = 0.23), and discharge MR severity (*p* = 0.11) did not show a significant difference in survival.

### 4.3. The Evolution of Mitral Valve Regurgitation After TAVI

Serial echocardiography showed a sustained shift from more-than-moderate MR at baseline toward lower grades after TAVI, with significant improvement persisting up to five years in patients with available follow-up. These findings are consistent with prior reports of early MR reduction and continued evolution beyond discharge [8,9,10]. MR regression was mechanism-dependent: functional MR improved to a greater extent than degenerative MR, supporting the concepst of afterload reduction and reverse remodeling as primary drivers of functional MR improvement [4,7,10]. Brown et al. demonstrated substantial MR regression by one year in patients with ≥moderate MR, whereas Baudo et al. reported heterogeneous trajectories among patients with untreated moderate MR, including improvement, stability, and progression [17,18]. Our cohort differs by restricting inclusion to more-than-moderate MR and by extending serial echocardiographic observation beyond the first year. However, the limited number of patients with degenerative MR, particularly at long-term follow-up, restricts robust comparison between etiologies. Our findings are compatible with a mechanism-aware staged-treatment framework, specifically in a cohort restricted to severe MR [4,6,10,15].

### 4.4. High-Risk Cohort

Importantly, our cohort represents a particularly high-risk population with severe aortic stenosis, significant concomitant MR, and substantial comorbidity. The observed decline in survival underscores these patients’ vulnerability and limits the number available for longitudinal assessment. Although MR improved in most patients, overall prognosis remained poor, reflecting a clinically advanced and comorbidity-rich population. Most patients were markedly symptomatic (NYHA III 77.7%, NYHA IV 16.1%) with intermediate-to-high procedural risk (EuroSCORE II 6.8 ± 5.1; STS 6.4 ± 4.8). In-hospital mortality was 11.6%, and survival showed continued attrition over 5 years, consistent with substantial competing risk in advanced bivalvular, global myocardial disease, already described by prior studies [11,12,13]. Nevertheless, the observed MR regression among patients with available follow-up provides descriptive insight into the evolution of concomitant MR following TAVI in this clinically challenging population.

### 4.5. Survival Based on Mitral Valve Regurgitation Improvement

We observed no statistically significant association between early MR improvement and 5-year survival. This aligns with the ongoing debate in the literature. While persistent moderate-to-severe MR after TAVI is commonly associated with worse outcomes, whether MR improvement independently drives survival remains uncertain [7,11,13,25]. Our results do not establish the lack of prognostic relevance of early MR improvement, due to a limited cohort. Rather, they indicate that, in a higher-grade MR cohort with substantial competing risk, early MR change may be an important hemodynamic response marker, but not a reliable surrogate for long-term survival [14,19,20].

### 4.6. Survival Based on Mitral Valve Regurgitation Etiology

In our cohort, no statistically significant survival difference according to MR etiology was demonstrated. However, substantial uncertainties remain, as the degenerative subgroup was small. Prior work has suggested that degenerative MR persists more frequently, and has been associated with less favorable clinical outcomes [7,9,10]. However, the findings are not uniform across cohorts, and may depend on patient selection and the degree of residual MR after TAVI [3,7,11]. Our findings should be considered preliminary, and emphasize the need for larger, mechanism-stratified higher-grade MR datasets.

### 4.7. Survival Based on Mitral Valve Regurgitation-Discharge Severity

Residual MR severity at discharge was not significantly associated with five-year survival in the global analysis, while confidence is limited by subgroup size. Although previous studies have associated residual higher-grade MR with adverse outcomes, our findings did not demonstrate a significant difference [4,11,19]. This likely reflects the limited sample size and exploratory nature of the analysis, and highlights the limitation of single-timepoint MR grading, particularly without comprehensive mechanism phenotyping. Baseline TR severity and LVEF categories were not statistically significantly associated with survival in this analysis, whereas a severely compromised functional status (NYHA IV) was associated with worse survival. These exploratory, unadjusted analyses do not establish the absence or independent presence of prognostic effects. Overall, these additional findings rather underscore the fact that the multidimensional nature of risk in mixed-valve disease is not fully captured by single parameters [8,13,15,26].

### 4.8. Clinical Translation

Clinically, these results are consistent with a staged strategy, as TAVI is frequently accompanied by meaningful MR regression, particularly in functional MR, and improvement may continue beyond discharge [4,5]. However, the observational design does not establish the superiority of this strategy or determine which patients can safely defer mitral intervention. Prognostication after TAVI in more-than-moderate MR patients requires systematic and closer follow-up, integrated phenotyping (valvular mechanisms, remodeling reserve, and right-heart involvement), and systemic comorbidity assessment, before deciding on adjunctive mitral procedures [27]. Patients with persistent higher-grade MR may benefit from optimization of heart failure therapy and Heart Team discussion regarding adjunctive mitral interventions when symptoms persist or MR remains prognostically relevant [6,16].

### 4.9. Limitations

This study had several limitations. First, due to its retrospective single-center design, small sample size, selected study population, and changes in TAVI era practice across the long inclusion period, the generalizability is limited. Although all patients underwent interdisciplinary Heart Team evaluation, individual determinants of treatment allocation were not captured as standardized research variables throughout the enrollment period. Changes in patient selection, device technology, procedural technique, and peri-procedural management likely contributed to clinical heterogeneity, and limit direct extrapolation to contemporary practice. Access route and valve platform were not included in the predefined dataset, and were therefore not analyzed retrospectively. Mid-term echocardiographic assessment was restricted to 45 surviving patients with available follow-up, introducing potential survivor and selection bias. Patients with more advanced disease or persistent MR may have been less likely to survive to follow-up, potentially leading to an overestimation of long-term MR improvement. Accordingly, these findings should be interpreted as descriptive outcomes among evaluable survivors, rather than representative of the entire baseline cohort. Exclusion of echocardiographic observations after subsequent mitral intervention was necessary to preserve assessment of the untreated course of MR. However, this approach may selectively remove patients with persistent or progressive MR from later time points and thereby contribute to an overestimation of favorable long-term MR evolution. Follow-up examinations were performed according to clinical routine, rather than at standardized prespecified intervals. MR severity was assessed during routine clinical care, rather than by an independent core laboratory. Additionally, some variability in MR grading cannot be excluded in the setting of changing loading conditions after TAVI and residual aortic regurgitation in a subset of patients. Several analyses were limited by small subgroup sizes, especially for degenerative MR and late follow-up cohorts, reducing statistical power and certainty of negative findings. Consequently, non-significant associations between survival and MR etiology, MR improvement, and residual MR grade should be considered exploratory, rather than definitive. Survival analyses according to discharge MR severity and early MR improvement were not performed using a prespecified landmark or time-dependent approach. These limitations also precluded reliable multivariable analysis of predictors of MR regression or mortality. Finally, heart-failure rehospitalization and validated quality-of-life measures were not systematically assessed. Likewise, secondary MR was not further classified into atrial and ventricular subtypes, because the retrospective study period did not provide sufficient measurements for reliable retrospective phenotyping throughout the cohort, limiting a more detailed mechanism-specific interpretation of MR regression after TAVI. Prospective multicenter studies with standardized imaging are needed to clarify the prognostic role of early MR change, discharge MR grade, and MR phenotype beyond established clinical covariates.

## 5. Conclusions

In patients with severe aortic stenosis, concomitant more-than-moderate MR, and with available mid-term echocardiographic follow-up, the regurgitation decreased consistently after TAVI. Despite these favorable dynamics, exploratory analyses did not demonstrate significant survival advantages according to MR etiology, early MR improvement, or discharge MR severity over five years, underscoring competing risks in advanced multivalvular disease. However, given the incomplete follow-up, the small and heterogeneous cohort, and the absence of multivariable adjustment, the survival findings should be interpreted as hypothesis-generating, rather than definitive evidence of absent prognostic effect. In summary, the findings are compatible with a staged, surveillance-first strategy with systematic reassessment and individualized Heart Team consideration of adjunctive mitral therapy for persistent symptomatic higher-grade MR. Prospective multicenter studies with mechanism-based MR phenotyping and cofactor identification are needed to clarify when staged adjunctive mitral therapies could translate into clinically meaningful benefit after TAVI.

## Figures and Tables

**Table 1:** Baseline clinical and echocardiographic characteristics.

	Total *n* = 112
Female gender, *n* (%)	63 (56.3)
Age, years (mean ± SD)	82 (±6.4)
BMI (mean ± SD)	25.6 (±5)
NYHA class I, *n* (%)	0 (0.0)
NYHA class II, *n* (%)	6 (5.4)
NYHA class III, *n* (%)	87 (77.7)
NYHA class IV, *n* (%)	18 (16.1)
Euroscore II (mean ± SD)	6.8 (±5.1)
STS (mean ± SD)	6.4 (±4.8)
Functional MR, *n* (%)	94 (84.0)
Degenerative MR, *n* (%)	18 (16.1)
Creatinine (mean ± SD), mg/dL	1.4 (±1.1)
COPD, *n* (%)	15 (13.4)
Stroke, *n* (%)	16 (14.3)
Atrial fibrillation, *n* (%)	55 (49.1)
Pacemaker, *n* (%)	4 (3.6)
CAD, *n* (%)	57 (50.9)
Previous cardiac procedure, *n* (%)	10 (8.9)
Previous PCI, *n* (%)	27 (24.1)
Previous CABG, *n* (%)	9 (8.0)
	
LVEF > 50, *n* (%)	57 (50.9)
LVEF 35–50, *n* (%)	41 (36.6)
LVEF < 35, *n* (%)	14 (12.5)
LVEDd, mm (mean ± SD)	50 (±9.0)
RV enlargement, *n* (%)	23 (20.5)
MS > mild, *n* (%)	6 (5.4)
Moderate–severe MR, *n* (%)	62 (55.4)
Severe MR, *n* (%)	50 (44.6)
AR > mild, *n* (%)	23 (20.5)
TR > mild, *n* (%)	65 (58.0)

BMI = body mass index, STS = Society of Thoracic Surgeons Risk Score, COPD = chronic obstructive pulmonary disease, CAD = coronary artery disease, PCI = percutaneous coronary intervention, CABG = coronary artery bypass graft, LVEF = left ventricular ejection fraction, LVEDd = left ventricular end-diastolic diameter, RV = right ventricle, MS = mitral stenosis, MR = mitral regurgitation, AR = aortic regurgitation, TR = tricuspid regurgitation. Values are presented as *n* (%) or mean ± SD. Percentages are based on available data.

**Table 2:** Echocardiographic data post-procedure.

	Total *n* = 107
LVEF > 50, *n* (%)	56 (52.3)
LVEF 35–50, *n* (%)	37 (34.6)
LVEF < 35, *n* (%)	11 (10.3)
LVEDd, mm (mean ± SD)	49 (±9.0)
RV enlargement, *n* (%)	25 (22.3)
MS > mild, *n* (%)	1 (0.9)
Mild MR, *n* (%)	15 (14)
Mild-Moderate MR, *n* (%)	15 (14)
Moderate MR, *n* (%)	24 (22.4)
Moderate-Severe MR, *n* (%)	29 (27.1)
Severe MR, *n* (%)	24 (22.4)
AR > mild, *n* (%)	15 (13.4)
TR > mild, *n* (%)	43 (38.4)

LVEF = left ventricular ejection fraction, LVEDd = left ventricular end-diastolic diameter, RV = right ventricle, MS = mitral stenosis, MR = mitral regurgitation, AR = aortic regurgitation, TR = tricuspid regurgitation. Values are presented as *n* (%) or mean ± SD. Percentages are based on available data.

**Table 3:** Overall reintervention and death statistics.

	Total *n* = 112
mTEER, *n* (%)	6 (5.4)
TMVI, *n* (%)	2 (1.8)
MVR, *n* (%)	2 (1.8)
AVR, *n* (%)	1 (0.9)
CABG, *n* (%)	1 (0.9)
Intrahospital death, *n* (%)	13 (11.6)
CV death, *n* (%)	21 (18.8)
Non-CV death, *n* (%)	49 (43.8)
Unknown cause of death, *n* (%)	42 (37.5)
Duration to death, years (mean ± SD)	2.6 (±2.7)

mTEER = mitral transcatheter edge-to-edge repair, TMVI = transcatheter mitral valve implantation, MVR = mitral valve replacement, AVR = aortic valve replacement, CABG = coronary artery bypass graft, CV = cardiovascular. Observational period from 2007–2024. Values are presented as *n* (%) or mean ± SD. Percentages are based on available data.

**Table 4:** Clinical and echocardiographic follow-up data.

	Total *n* = 45
Follow-up, years (mean ± SD)	4.18 (±2.06)
NYHA class I, *n* (%)	10 (22.2)
NYHA class II, *n* (%)	14 (31.1)
NYHA class III, *n* (%)	14 (31.1)
NYHA class IV, *n* (%)	7 (15.6)
Atrial fibrillation, *n* (%)	20 (44.4)
Pacemaker, *n* (%)	14 (31.1)
	
LVEF > 50, *n* (%)	23 (51.1)
LVEF 35–50, *n* (%)	18 (40.0)
LVEF < 35, *n* (%)	4 (8.9)
LVEDd, mm (mean ± SD)	51.0 (±7.1)
RV enlargement, *n* (%)	14 (31.1)
MS > mild, *n* (%)	3 (6.7)
No MR, *n* (%)	4 (8.9)
Mild MR, *n* (%)	16 (35.6)
Mild–moderate MR, *n* (%)	6 (13.3)
Moderate MR, *n* (%)	10 (22.2)
Moderate–severe MR, *n* (%)	6 (13.3)
Severe MR, *n* (%)	3 (6.7)
AR > mild, *n* (%)	9 (20.0)
TR > mild, *n* (%)	14 (31.1)

LVEF = left ventricular ejection fraction, LVEDd = left ventricular end-diastolic diameter, RV = right ventricle, MS = mitral stenosis, MR = mitral regurgitation, AR = aortic regurgitation, TR = tricuspid regurgitation. Follow-up defined as last echocardiography performed after a period of one year. Values are presented as *n* (%) or mean ± SD. Percentages are based on available data.

## Data Availability

Due to privacy restrictions, raw data cannot be provided as Supplementary Material, but de-identified data are available from the corresponding author upon reasonable request.

## Supplementary Materials

The following supporting information can be downloaded at: https://www.mdpi.com/article/10.3390/jcm15218259/s1, Figure S1: 5-year survival based on mitral regurgitation improvement in the functional cohort; Figure S2: 5-year survival based on LVEF category; Figure S3: 5-year survival based on TR severity; Figure S4: 5-year survival based on NYHA class.

## Abbreviations

The following abbreviations are used in this manuscript:

TAVI	[Transcatheter aortic valve implantation]
MR	[Mitral valve regurgitation]
AS	[Aortic valve stenosis]
NYHA	[New York Heart Association]
LVEF	[Left ventricular ejection fraction]
TR	[Tricuspid valve regurgitation]
TTE	[Transthoracic echocardiography]
SD	[Standard deviation]
BMI	[Body Mass Index]
STS	[Society of Thoracic Surgeons Risk Score]
COPD	[Chronic obstructive pulmonary disease]
CAD	[Coronary artery disease]
PCI	[Percutaneous coronary intervention]
CABG	[Coronary artery bypass grafting]
LVEDd	[Left ventricular end-diastolic diameter
RV	[Right ventricle]
MS	[Mitral stenosis]
AR	[Aortic regurgitation]
mTEER	[Mitral valve Transcatheter Edge-to-Edge Repair]
TMVI	[Transcatheter Mitral Valve Implantation]
MVR	[Mitral valve replacement]
CV	[Cardiovascular]
