# Supplementary material for: The Fate of Mitral Valve Regurgitation After TAVI
Source: J Clin Med. 2026 Oct 23;15(21):8259. doi: 10.3390/jcm15218259 (PMC13763798; doi:10.3390/jcm15218259)
Supplement: Supplementary file 1 [file jcm-15-08259-s001.zip › jcm-4615828-supplementary.pdf]

The fate of Mitral valve regurgitation after TAVI

Supplemental Material

1. Supplemental Figure S1, Page 2
2. Supplemental Figure S2, Page 3
3. Supplemental Figure S3, Page 4
4. Supplemental Figure S4, Page 5

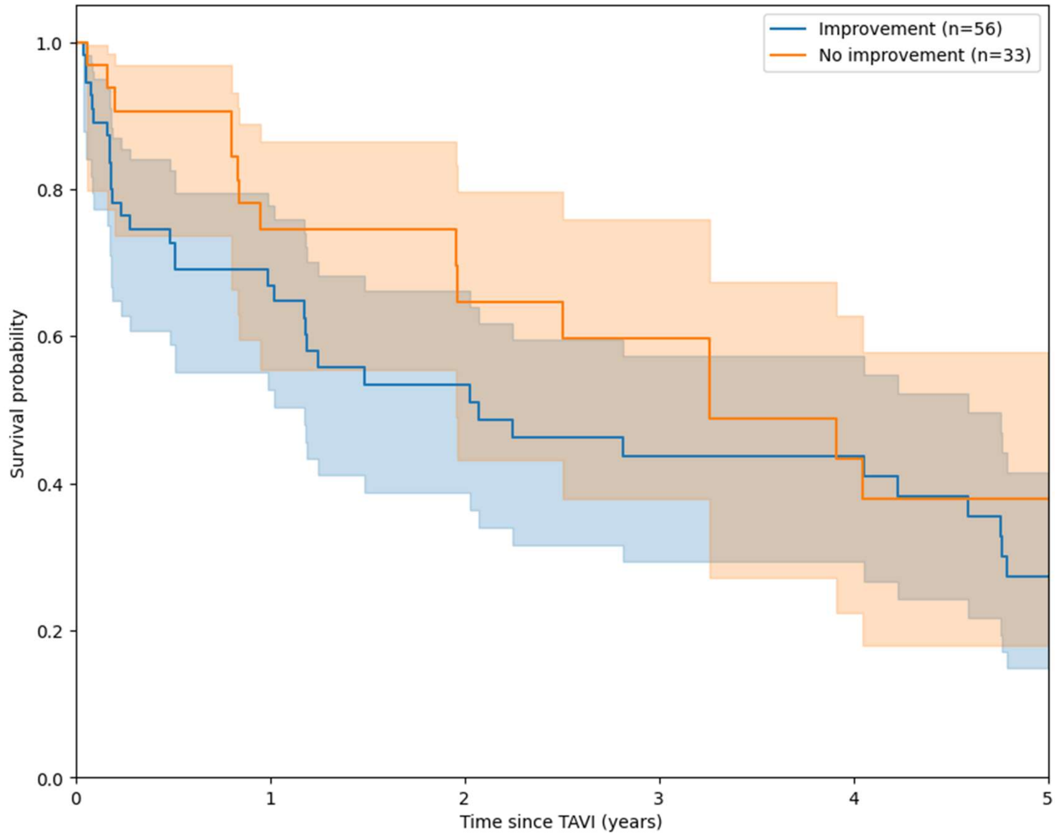

Improvement (n=56)						
At risk	56	31	22	17	16	0
Censored	0	7	10	11	12	22
Events	0	18	24	28	28	34
No improvement (n=33)						
At risk	33	20	13	12	8	0
Censored	0	5	10	10	11	18
Events	0	8	10	11	14	15

Supplemental Figure S1. Kaplan-Meier curves comparing 5-year survival based on mitral regurgitation improvement in the functional cohort at discharge after TAVI. Shaded areas indicate 95% confidence interval, x-axis shows years since procedure, y-axis shows survival probability. Log-rank p=0.12.

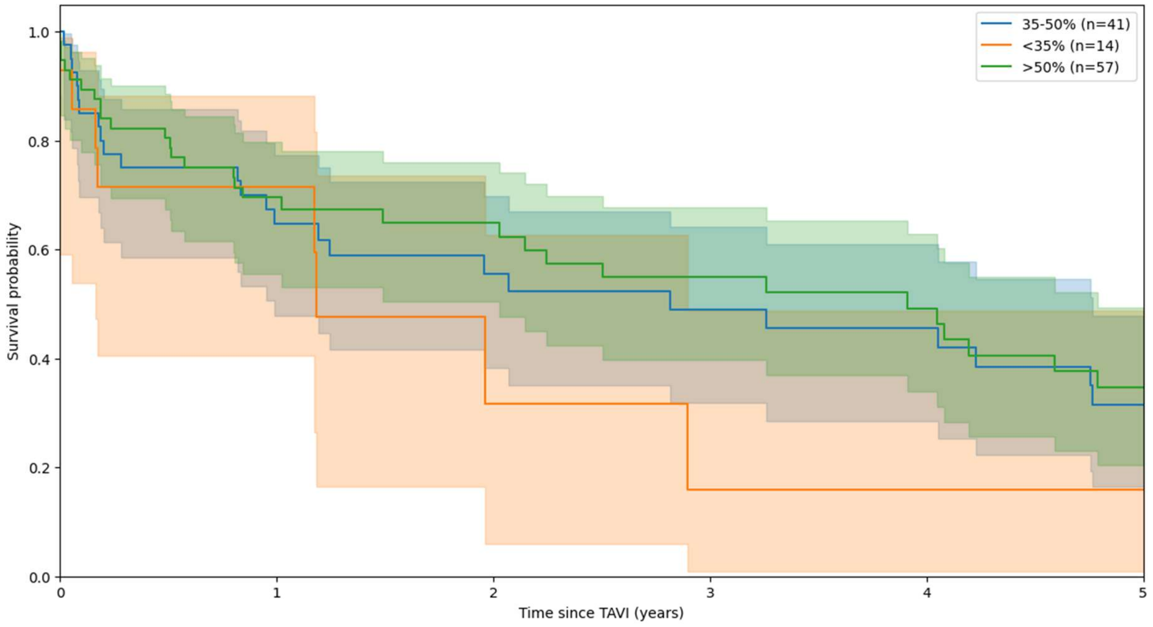

	0	1	2	3	4	5
35-50% (n=41)						
At risk	41	24	17	15	13	0
Censored	0	3	7	7	8	17
Events	0	14	17	19	20	24
<35% (n=14)						
At risk	13	7	2	1	1	0
Censored	0	3	5	5	5	6
Events	1	4	7	8	8	8
>50% (n=57)						
At risk	56	31	26	20	17	0
Censored	0	9	12	14	15	27
Events	1	17	19	23	25	30

Supplemental Figure S2. Kaplan-Meier curves comparing 5-year survival based on left ventricular ejection fraction (LVEF) category at baseline. Shaded areas indicate 95% confidence interval, x-axis shows years since procedure, y-axis shows survival probability.

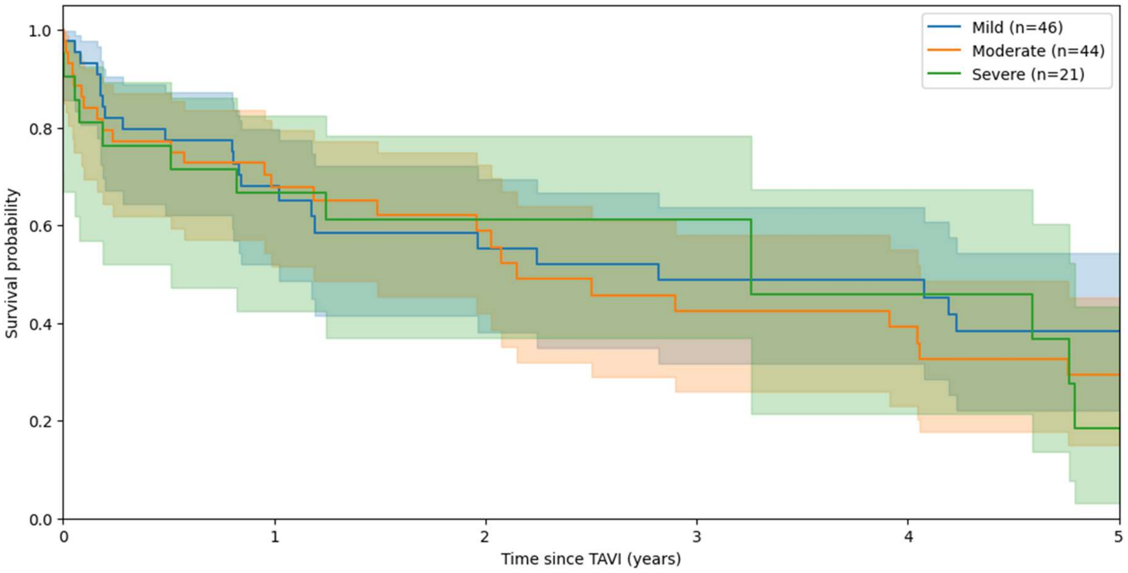

	0	1	2	3	4	5
**Mild (n=46)**						
At risk	45	23	17	14	14	0
Censored	0	9	11	12	12	23
Events	1	14	18	20	20	23
**Moderate (n=44)**						
At risk	44	27	18	13	12	0
Censored	0	3	9	9	9	18
Events	0	14	17	22	23	26
**Severe (n=21)**						
At risk	20	12	10	9	5	0
Censored	0	2	3	4	6	8
Events	1	7	8	8	10	13

Supplemental Figure S3. Kaplan-Meier curves comparing survival based on tricuspid regurgitation (TR) severity at baseline. Shaded areas indicate 95% confidence interval, x-axis shows years since procedure, y-axis shows survival probability.

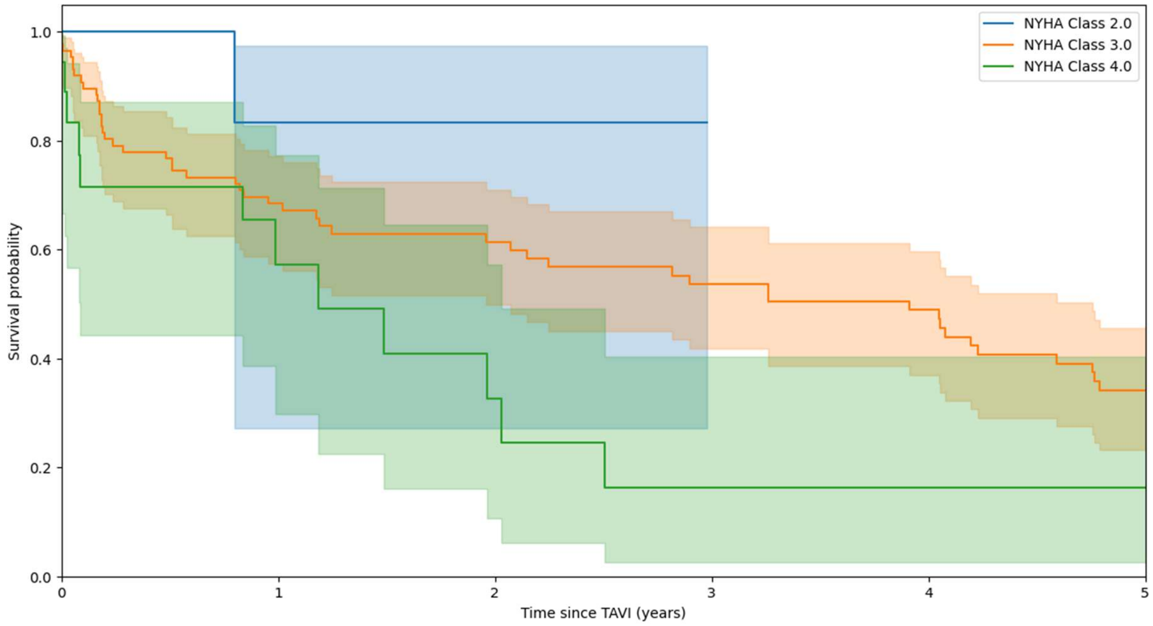

	0	1	2	3	4	5
**NYHA Class 2.0**						
At risk	6	3	1	0	0	0
Censored	0	2	4	5	5	5
Events	0	1	1	1	1	1
**NYHA Class 3.0**						
At risk	85	52	40	34	30	0
Censored	0	8	15	16	17	38
Events	2	27	32	37	40	49
**NYHA Class 4.0**						
At risk	18	7	4	2	1	0
Censored	0	4	4	4	5	6
Events	0	7	10	12	12	12

Supplemental Figure S4. Kaplan-Meier curves comparing 5-year survival based on NYHA class at baseline. Shaded areas indicate 95% confidence interval, x-axis shows years since procedure, y-axis shows survival probability.
